# Comparative review of copper-associated chronic hepatitis in dogs and Wilson disease in humans

**DOI:** 10.3389/fvets.2025.1692603

**Published:** 2025-12-04

**Authors:** Tarini Ullal, Eunju April Choi, Dorina Gui, Valentina Medici

**Affiliations:** 1 Department of Medicine and Epidemiology, School of Veterinary Medicine, University of California, Davis, Davis, CA, United States; 2 Department of Pathology, Microbiology, and Immunology, School of Veterinary Medicine, University of California, Davis, Davis, CA, United States; 3 Department of Pathology and Laboratory Medicine, School of Medicine, University of California, Davis, Sacramento, CA, United States; 4 Division of Gastroenterology and Hepatology, Department of Internal Medicine, University of California, Davis, Sacramento, CA, United States

**Keywords:** copper, liver, chronic hepatitis, Wilson disease, penicillamine, chelator, ATP7B, ceruloplasmin

## Abstract

Copper-associated chronic hepatitis (CAH) in dogs and Wilson disease (WD) in humans are progressive hepatic disorders caused by copper accumulation. Although both diseases share similar pathomechanisms of copper-induced liver injury, they diverge in some aspects of etiology, clinical manifestations, diagnostic methods, and therapeutic strategies. Wilson disease arises from inherited ATP7B mutations while CAH in dogs might be more influenced by excess dietary copper with ATP7B mutations playing a lesser role. Dogs exhibit hepatic disease whereas humans show hepatic and/or extrahepatic manifestations, including neuropsychiatric and ocular signs. Dogs with CAH accumulate copper centrilobularly unlike human patients who develop copper periportally initially. There are several non-invasive diagnostic tools used to monitor the effect of treatment in humans with WD that are not available for dogs with CAH. Copper chelation and dietary copper restriction are used to treat dogs with CAH and humans with WD, but liver transplantation is not an option for dogs with CAH. This comparative review highlights the similarities and differences between CAH and WD, offering insights that may improve our understanding and management of copper associated liver diseases in dogs and human patients.

## 1 Introduction

Copper is an essential trace element required for numerous physiological processes in mammals (1, 2). These processes involve copper-dependent enzymes that use copper as a cofactor, including cytochrome c oxidase for electron transport chain function and ATP production, superoxide dismutase for antioxidant defense, lysyl oxidase for collagen and elastin formation, dopamine hydroxylase for neurotransmitter synthesis, tyrosinase for pigmentation, sulfhydryl oxidase for keratinization, and ceruloplasmin and hephaestin for iron homeostasis. Maintenance of copper homeostasis is vital because both copper deficiency and excess can result in significant pathology. Specifically in the case of excess copper, there is heightened potential for free radical formation, oxidative damage, mitochondrial stress, and ultimately cell death driven by copper, known as cuproptosis (3).

Copper metabolism is highly preserved between mammalian species and tightly regulated to prevent imbalances. Copper is absorbed at the intestines via copper transport 1 protein (CTR1) and divalent metal transporter 1 (DMT1). Once absorbed into enterocytes, copper is immediately bound to glutathione, metallothionein (MT), or chaperones to prevent oxidative damage. Copper chaperone antioxidant 1 (ATOX1), escorts copper to ATP-ase copper transporting alpha (ATP7A), which then exports copper to the systemic circulation.

Circulating copper enters hepatocytes via CTR1. Once intracellular, copper binds to glutathione, metallothionein, or copper chaperone proteins to prevent oxidative damage and to incorporate copper in processes such as mitochondrial oxidative phosphorylation. Copper chaperone antioxidant 1 (ATOX1) presents copper to ATP-ase copper transporting beta (ATP7B), which incorporates copper into apo-ceruloplasmin in the trans-Golgi network to form holo-ceruloplasmin and facilitate copper excretion from the liver. The copper-ceruloplasmin complex also mediates iron oxidation and binding of iron to ferritin (4). When copper is elevated, ATP7B facilitates biliary excretion of copper with the aid of Copper Metabolism Murr1 Domain-containing protein 1 (COMMD1) (1, 2). The ATP7A transporter can also facilitate hepatic copper excretion (5).

Both dogs and humans can be affected by disorders of excess hepatic copper. These disorders are known as copper-associated chronic hepatitis (CAH) in dogs and Wilson disease (WD) in humans. Pathologic levels of hepatic copper cause hepatocellular injury through the production of reactive oxygen species (ROS), mitochondrial dysfunction, and inflammation, which ultimately leads to fibrosis and cirrhosis (3, 6–8). Copper-associated chronic hepatitis (CAH) in dogs and WD in humans share pathophysiology, but they differ in some aspects of etiology, clinical presentation, diagnostic tools, and treatment approaches (Table 1) (7, 9–47). Table 1 summarizes the comparative features of CAH in dogs and WD in humans.

**Table 1:** Main features of copper-associated chronic hepatitis in dogs and Wilson disease in humans.

	Dogs with copper-associated chronic hepatitis (CAH)	Humans with Wilson disease (WD)
Prevalence	1/3 of dogs with chronic hepatitis have copper associated chronic hepatitis (9) Increased prevalence since 1990’s (10, 11)	1:30,000 to 1:50,000 (12) Hepatic and neurologic forms most common (7)
Demographic features		
Age at onset	Generally middle age to older (13, 14)	Variable; typically between 2^nd^ and 4^th^ decade of life (15)
Sex	Females in Labrador retrievers (16) and Doberman pinschers (17)	Males and females both affected (18)
Breed/Ethnicity	Labrador retrievers (16) Doberman pinschers (17) Bedlington terriers (19) Dalmatians (20, 21) West Highland white terriers (22) Non-predisposed breeds as well (11)	Occurs worldwide (7)
Clinical manifestations		
Genotype–Phenotype correlation	*COMMD1* mutation established in Bedlington terriers (23) *ATP7B* and *ATP7A* mutations implicated in Labrador retrievers (24, 25)	Hundreds of identified *ATP7B* mutations, incomplete penetrance (26); poor genotype–phenotype correlation (27)
Hepatic	Ranges from subclinical hepatopathy to liver failure (13)	Ranges from subclinical hepatopathy to acute or chronic liver failure (7)
Neurologic/behavioral	None	Copper deposition in basal ganglia resulting in neuropsychiatric disease (15)
Ocular	No ocular abnormalities	Kayser-Fleischer rings; sunflower cataracts (28); damage to nerves in corneal nerve plexus (7)
Hemolytic anemia	Rare, but documented in Bedlington terrier (29)	Coombs negative hemolytic anemia (30)
Joint	None	Osteomalacia, osteoporosis, arthritis, or arthralgia (30)
Renal	Fanconi’s syndrome (31)	Renal tubular dysfunction (30)
Cardiac	None	Cardiac arrhythmias, cardiomyopathy, rhabdomyolysis (30)
Dietary involvement		
Dietary copper	Increased dietary copper content and bioavailability (1, 32, 33)	Introduction of higher copper foods can contribute to progression (7)
Laboratory findings and measures of copper		
Liver enzymes and function parameters	Transaminase activity +/− liver function abnormalities (13)	Transaminase activity +/− liver function abnormalities (7)
Serum copper	Does not correlate with hepatic copper (13, 34) Increases with inflammatory conditions (35)	Low serum copper because of reduced ceruloplasmin, but can be normal to elevated from elevated exchangeable copper (7)
Ceruloplasmin	Normal to elevated in dogs with CAH (36)	< 20 mg/dL or lower consistent with WD, but many limitations (37)
Urinary copper	Urine copper:zinc ratio associated with hepatic copper, but overlap with healthy Labrador retrievers (38)	Elevated 24-h urinary copper indicative of WD; >1x or >2x upper limit of normal; > 1.6 μmol/L or > 100 μg/24 h in symptomatic patients (39–41)
Non ceruloplasmin bound copper	Not evaluated in dogs	Maintenance: 5–15 μg/dL; Overtreatment: < 5 μg/dL; Treatment failure: > 15 μg/dL (42)
Hepatic copper	Normal value: 200 to 400 μg/g dry weight; > 1,000 μg/g concerning for CAH (8)	Normal value: 20 to 50 μg/g dry weight; > 250 μg/g dry weight concerning for WD (43)
Liver histology		
Chronic hepatitis to fibrosis and cirrhosis	Positive rhodanine stain in centrilobular hepatocytes progressing to midzonal and then panlobular (13); Rhodanine stained positive pigment granulomas with chronic hepatitis progressing to fibrosis and cirrhosis (44); Mitochondrial changes including shrunken nuclei and accumulation of electron dense material (45, 46)	Rhodanine staining has poor sensitivity; copper accumulation is periportal (47); Hepatic steatosis, spotty hepatocyte necrosis and lymphocytic hepatitis that progresses to fibrosis and cirrhosis (48); Mallory-Denk bodies; glycogenated nuclei (15, 48); Ultrastructural mitochondrial abnormalities (49)
Steatosis	Not observed	Moderate to severe steatosis (50)

This review aims to compare and contrast CAH and WD. Highlighting the similarities and differences could provide translational insights that guide and improve diagnostic and therapeutic strategies for both dog and human patients.

## 2 Epidemiology and signalment of copper-associated chronic hepatitis (CAH) and Wilson disease (WD)

Copper associated chronic hepatitis (CAH) is a common cause of liver disease in dogs that is estimated to affect one-third of dogs with chronic hepatitis (9). A study from a single institution over the 10-year period (2010–2020) corroborated that elevated hepatic copper is a common problem in dogs. Of 4,559 dogs that had undergone a liver biopsy and hepatic copper quantification, 50 and 19% of them had copper concentrations above the normal reference interval (200 to 400 μg/g dry weight) and > 1,000 μg/g dry weight, respectively. Dogs can be diagnosed at any age, but are usually diagnosed when middle-aged to older although subclinical disease can occur sooner (13, 14). Breeds such as Labrador retrievers (10), West Highland white terriers (48), Doberman pinschers (49), Dalmatians (21), and Bedlington terriers (19) are predisposed to CAH compared to other breeds (11). Females are more commonly affected in the Labrador retrievers (10, 24) and Doberman pinschers (17). In both non-predisposed and especially in predisposed breeds, higher hepatic copper concentrations and an increased prevalence of CAH have been observed since the 1990’s possibly because of changes in the nutrient guidelines for commercial dog foods (10, 11). In 1997, the Association of American Feed Control Officials recommended discontinuing the use of cupric oxide in commercial dog foods in place of more bioavailable forms such as copper sulfate. Additionally, the limit set for maximum copper concentration in adult maintenance dog diets was eliminated in 2007. This has permitted commercial dog foods to incorporate as much copper in the diet as desired without any regulation and has potentially contributed to the rising prevalence of CAH in dogs.

Wilson disease (WD) is a rare autosomal recessive disorder affecting 1 in 30,000 to 50,000 individuals worldwide. However, the prevalence might be underestimated because of clinical heterogeneity and limited awareness. In general, the clinical prevalence is considered lower than the genetic prevalence (12). The common age of onset is in adolescence and young adulthood to middle age, but the range of years affected can be wide. Hepatic presentations of WD occur earlier in age while neurologic symptoms present later in adulthood (7). Both females and males are equally affected, but hepatic presentation is more common in females and the neurologic presentation is more common in males (18).

## 3 Genetics and environmental factors involved in copper-associated chronic hepatitis (CAH) and Wilson disease (WD)

In dogs with CAH, the genetic landscape is less known compared to humans with WD, but ATP7B dysfunction has been implicated in several breeds (24, 50–52). CAH was first recognized in the Bedlington terrier and linked to a 13 kb autosomal recessive deletion in the exon 2 of the *COMMD1* gene. Selective breeding reduced, but did not eliminate CAH in the Bedlington terrier or other breeds, suggesting other contributing factors (51, 53, 54). A genome wide association study in 235 Dutch Labrador retrievers from samples collected in the early 2000s identified a missense mutation in *ATP7B (ATP7B:c.4358G > A)* causing *ATP7B* mislocalization in the endoplasmic reticulum instead of the trans-Golgi network and higher hepatic copper. Similar associations with *ATP7B* were reported in other breeds such as the Doberman pinscher, Cavalier King Charles spaniel, and *COMMD1*^+/+^ Bedlington terriers. The *ATP7B* effect is additive such that homozygous mutants have higher hepatic copper concentrations than heterozygous individuals (24, 50–52). However, the *ATPB* variant allele is not fully predictive or diagnostic for CAH. In a study of samples retrieved between 2013 and 2021 from a single university laboratory database, 25% (7/28) of healthy Labrador retrievers dogs carried the variant allele and nearly 50% (21/45) of Labrador retrievers with elevated hepatic copper lacked the variant allele (25). Also, only 8.3% of the heritable variation in hepatic copper concentrations in 235 Dutch Labrador retrievers could be attributed to the *ATP7B* mutation (24). Other genetic modifiers also affect copper concentration such as *ATP7A* and resistin (*RETN*) genes, which negatively correlate with hepatic copper in Labrador retrievers (55). There is also strong evidence to support that environmental factors, such as diet, impact hepatic copper concentrations given that hepatic copper concentrations have increased ever since AAFCO guidelines were amended (33, 56). Dietary factors may have a greater impact on dogs than humans because dogs are fed standardized, commercially formulated diets, whereas humans consume a more varied and individualized diet. Additionally, the effects of genetic predispositions in dogs are likely amplified by inbreeding. The impact of these factors could explain some of the clinical differences between CAH and WD.

In humans, WD arises from mutations in the *ATP7B* gene located on chromosome 13q14. Hundreds of pathogenic variants have been identified according to the Human Gene Mutation Database, and not all mutations are causative of copper accumulation. Higher genetic frequencies have been documented in Sardinia, parts of India, Pakistan, Romania, and the Middle East, correlating with increased carrier rates (12, 26). There is no predictable correlation between genotype and clinical phenotype (27). However, at the molecular level, common missense mutations have resulted in functional impairments or mislocalization of the mutated ATP7B (57). The missense mutation His1069Q on exon 14 is the most common in Europe and North America and the missense R778L on exon 8 predominates in East Asia (58). Given the poor genotype–phenotype correlation, WD is believed to be caused by a complex combination of genetic, metabolic, and epigenetic factors (27, 59–63). Modifying genes proposed to be associated with WD include esterase D (ESD), INO80 chromatin remodeling complex, apolipoprotein (APOE), methyl-CpG Binding Domain Protein 6 (MBD6), patatin-like phospholipase domain-containing 3 gene (PNPLA3), and methylenetetrahydrofolate reductase (MTHFR). Recent evidence indicates a possible role for prion protein in the pathogenesis of WD, where ATP7B dysfunction results in hepatic expression of prion protein, which in turns promotes hepatocyte copper accumulation (64). Differences in DNA methylation between patients with WD and other liver conditions support that unique epigenetic alterations occur in WD (65). Rodent models of WD demonstrate that not only excess copper, but also the maternal environment and nutritional factors such as betaine and choline supplementation can influence methionine metabolism and availability of methyl groups for DNA and histone methylation, resulting in differential methylation patterns (66–68). These external influences could contribute to the poor genotype–phenotype correlation and the wide phenotypic variability observed in human patients with WD.

## 4 Pathophysiology of copper-induced hepatic injury in copper-associated chronic hepatitis (CAH) and Wilson disease (WD)

Hepatic copper overload in CAH and WD overwhelms the buffering capacity of copper chaperones and metallothioneins. This results in excessive free copper that can participate in Haber-Weiss and Fenton reactions with consequent release of reactive oxygen species (69). Free radicals then damage the mitochondrial membrane, mitochondrial DNA structure, and energy transport chain, which leads to accelerated hepatocyte death (3, 6, 70, 71). Copper also directly triggers cell death by accumulating in mitochondria and activating pathways involving the mitochondrial ferredoxin 1 (FDX1) protein resulting in cuproptosis (72). Excess intracellular copper also activates autophagy via various mechanisms (73). Copper-induced dysregulation of nuclear receptors also is an important part of the pathogenesis of lipid dysmetabolism in human patients with WD (74, 75). Hepatic injury and death triggers inflammatory mediators (76), which result in acute or chronic hepatitis and activates hepatic stellate cells into myofibroblasts leading to fibrous deposition and ultimately cirrhosis (40, 77).

## 5 Clinical and laboratory features of copper-associated chronic hepatitis (CAH)

In dogs, CAH presents with subclinical disease in approximately one-fourth of patients. The diagnosis is pursued after detecting a predominantly hepatocellular enzymopathy (increased alanine aminotransferase (ALT) with or without aspartate aminotransferase (AST) levels), or a mixed pattern with elevations in alkaline phosphatase (ALP) and gamma-glutamyl transferase (GGT) as well. The complete blood count can show a non-regenerative, normocytic, normochromic anemia from an anemia of chronic disease (8). Rare reports of a copper-induced hemolytic anemia have been documented in the Bedlington terrier, but this clinical manifestation is uncommon (19, 29).

Clinical signs can initially be non-specific in nature and then progress to signs more specific of liver disease in later stages of disease. Examples are listed below.

Non-specific Signs:

- Hyporexia to anorexia
- Lethargy
- Vomiting
- Diarrhea
- Weight loss

Specific Signs:

- Icterus
- Abdominal distension from ascites
- Neurologic signs from hepatic encephalopathy
- Polydipsia/polyuria
- Melena from portal hypertension

In later stages of disease, elevated liver enzyme activity will generally be accompanied by abnormalities in liver function characterized by decreased albumin, cholesterol, blood urea nitrogen, increased bilirubin, and in the final stage, hypoglycemia (8). One of the unique manifestations of CAH is a Fanconi’s syndrome that develops from renal tubular epithelial copper accumulation. Glucosuria with or without proteinuria will be detectable on the urinalysis but typically resolves with treatment of CAH. Neurologic and ocular signs as a result of copper accumulation have not been observed in dogs with CAH.

## 6 Clinical and laboratory features of Wilson disease (WD)

In humans with WD, copper accumulates in various organs resulting in a wide potential range of symptoms including hepatic, neurologic, psychiatric, renal, cardiac, and skeletal manifestations. The most common clinical presentations are hepatic and/or neuropsychiatric disease. One of the early organs affected by WD is the liver. Patients with hepatic WD are initially asymptomatic with increased transaminase activity (AST > ALT), but like dogs with CAH, can progress to have non-specific and then more specific symptoms of liver disease. Examples of symptoms include:

- Fatigue
- Lack of appetite
- Jaundice
- Compensated or decompensated cirrhosis

Three to 5% of WD patients present in acute liver failure. Clinical signs and labwork abnormalities of patients in acute liver failure include (7, 78, 79):

- Low normal or normal ALP
- Jaundice, hyperbilirubinemia
- Non-immune hemolytic anemia
- Coagulopathy
- Ascites
- Encephalopathy
- Renal dysfunction

WD involves the central nervous system because of copper deposition, especially in the basal ganglia. Neuropsychiatric signs can include (80):

- Dysarthria (most common)
- Ataxia
- Dystonia (focal, segmental, or generalized)
- Tremors
- Seizures
- Dysphagia
- Parkinsonism
- Irregular dance-like movements (chorea)
- Mood instability
- Depression
- Sleep disorders
- Cognitive decline

Unlike dogs with CAH, human patients with WD can present with Kayser-Fleischer (KF) rings, which are dark, golden-brown rings at the edge of cornea that are detected by slit-lamp. The KF rings are not entirely specific for WD, but they are evident in virtually all patients with neurologic signs and symptoms. A less common manifestation is sunflower cataracts, which are located under the anterior lens capsule. Both ocular manifestations should resolve with treatment of WD (28). Patients with WD can also present with associated hematologic, cardiac, renal, skeletal, and endocrine manifestations including intravascular hemolysis, renal tubular dysfunction resulting in a Fanconi syndrome, hypokalemia, hypouricemia, skeletal or muscle weakness, cardiomyopathy, arrhythmias, atrial fibrillation, hypoparathyroidism, infertility, and frequent miscarriages (7).

## 7 Hypotheses to explain the clinical differences between copper-associated chronic hepatitis (CAH) and Wilson disease (WD)

Unlike human patients with WD, dogs do not develop ocular, neurologic, cardiac, or musculoskeletal signs as a result of copper accumulation. While increased copper concentrations in the cornea and brain have been documented in a few older Bedlington terrier dogs with CAH, none of the dogs were clinical for any ocular or neurologic signs (19, 36). Because the lifespan of dogs is much shorter than that of humans, there might not be adequate time to accumulate clinically significant copper concentrations in extrahepatic organs. Dogs with CAH might also succumb to their liver disease before substantial systemic copper accumulates given the progressive nature of the disease and because the diagnosis of CAH is often delayed until dogs are clinically ill. It is also possible that the degree of systemic copper accumulation is underestimated in dogs because copper is not routinely quantified or evaluated in tissues such as the brain or eyes. Clinical signs of neuropsychiatric disease could also be overlooked in dogs with CAH because it is challenging to diagnose dogs with anxiety, depression, mood, behavioral, or sleep disorders because dogs are unable to communicate their clinical signs. It also can be difficult to differentiate copper-induced neurologic disease from signs of hepatic encephalopathy.

Potential reasons for the differences in clinical manifestations and organ involvement between dogs CAH and humans with WD could be attributed to differing disease etiologies—particularly genetic predisposition and dietary factors. Dietary copper intake might play a much greater role in dogs with CAH relative to *ATP7B* mutations. Excess dietary copper contributes to hepatic overload of copper in dogs with CAH, but intact ATP7B function enables biliary excretion of copper to prevent copper accumulation in extrahepatic sites. The mutated ATP7B in human patients with WD compromises biliary excretion of copper and inhibits synthesis of holoceruloplasmin, which results in elevated circulating non-ceruloplasmin bound copper that is more capable of reaching extra-hepatic organs (81, 82). The absolute quantity of non-ceruloplasmin bound copper is overall measurably lower in dogs compared to humans, which further decreases the risk of extrahepatic copper accumulation in dogs (83). These differences in lifespan, detection and recognition, disease etiology, and circulating copper concentrations could explain the differences in clinical manifestations observed between dogs with CAH and humans with WD.

## 8 Diagnosis of copper associated chronic hepatitis (CAH)

**Figure 1:**
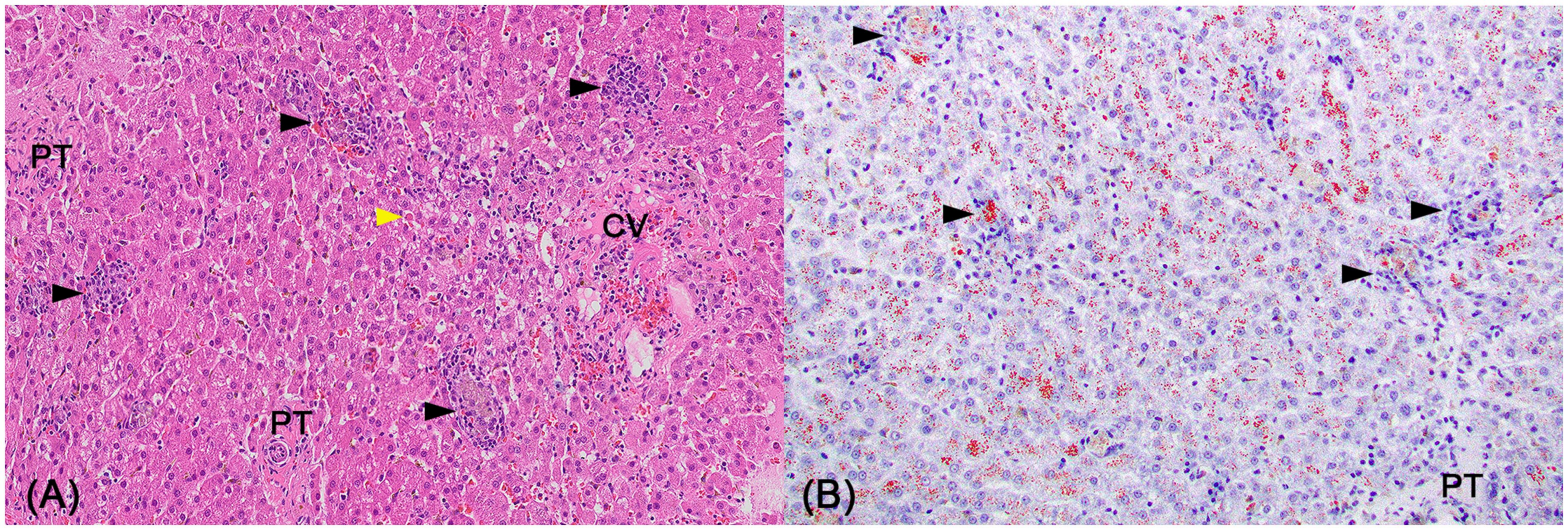
Liver histology of copper associated chronic hepatitis (CAH). **(A,B)** Liver histology at 200x magnification from an 8-year-old female spayed Labrador Retriever dog diagnosed with copper associated chronic hepatitis (CAH) with hepatic copper of 3,400 μg/g dry weight showing **(A)** the general increase in lymphocytes throughout, including the central vein (CV) adventitia, portal tract (PT) stroma, and sinusoids on the hematoxylin and eosin-stained section. Pigment granulomas (black arrowheads) are scattered and an individual necrotic hepatocyte is highlighted (yellow arrowhead). **(B)** This is a rhodanine-stained section highlighting the copper granules in a red-orange hue. Copper granules are found in hepatocytes and pigment granulomas (black arrowhead). The copper accumulation is nearly diffuse but slightly less in periportal hepatocytes compared to centrilobular and midzonal hepatocytes. Images obtained courtesy of Dr. Eunju April Choi.

The diagnostic evaluation of CAH in dogs begins with a detailed history, physical examination, complete blood count, biochemistry panel, and urinalysis. Ultimately, a liver biopsy to obtain histology and copper quantification is required for diagnosis. Biopsies should ideally be taken via laparoscopic or a laparotomy approach to optimize diagnostic quality (84–86). Histopathologic features consistent with CAH are copper accumulation in centrilobular hepatocytes, rhodanine grade ≥ 3/5, rhodanine staining positive pigment granulomas, lymphocytic to lymphohistiocytic hepatitis in areas of copper accumulation with multifocal scattered hepatocyte apoptosis to necrosis, and early fibrosis that can progress to panlobular copper distribution, chronic hepatitis, and cirrhosis (Figure 1). Bedlington terriers were the original model of CAH that demonstrated these histopathologic findings. Rhodanine and rubeanic staining highlighted cytoplasmic copper granules in hepatocytes. Electron microscopy and electron dispersion spectrophotometry revealed electron dense bodies in lysosomes consistent with sequestered copper (19, 87). Ultrastructural studies show that as hepatic copper concentrations increase and the lysosomal compartment is overwhelmed, copper accumulates in the nuclei and cytoplasm of hepatocytes (46), causing DNA and mitochondrial damage (variable sizes with loss of cristae) (45) likely via oxidative stress (6). Dogs with CAH usually have measurable copper concentrations > 1,000 μg/g dw, but there is no definitive cut-off for diagnosis and dogs with concentrations 600–1,000 μg/g dw can still have CAH (8). Conversely, Bedlington terriers with elevated hepatic copper > 1,000 μg/g dw might have minimal to no histologic evidence of hepatitis or injury (45) until they acutely develop acute hepatic necrosis and hemolytic anemia. Hepatic copper quantification is generally performed with flame atomic absorption spectroscopy (FAAS) or inductively coupled plasma mass spectrometry (ICP-MS). Hepatic copper can also be estimated using digital image analysis, which accounts for lobular variation in architecture and copper concentration to avoid underestimating copper concentrations (88). Serum copper levels are neither significantly increased nor correlated with hepatic copper concentrations and therefore are not used to diagnose CAH in dogs (19).

Other potential diagnostic biomarkers that require further study include urinary copper:zinc ratio, which was significantly associated with hepatic copper concentrations in Labrador retrievers, but there was overlap between the groups with elevated (> 400 μg/g dw) and normal hepatic copper. Erythrocyte copper chaperone for superoxide dismutase (CCS) levels and CCS/superoxide dismutase (SOD1) ratios were significantly decreased in Labrador retrievers with increased hepatic copper > 400 μg/g dw relative to Labrador retrievers with normal hepatic copper with no overlap (89). Another potential biomarker is microRNAs, which are small noncoding RNAs that regulate gene expression. Serum microRNA-122 was initially found to be significantly increased in Labrador retrievers with high hepatic copper concentrations (90), but this was later contradicted by a study in which serum cfa-miR-30b was significantly upregulated in Labrador retrievers with elevated hepatic copper levels (> 400 μg/g dw) relative to Labrador retrievers with normal hepatic copper (91). Hepatic copper quantification paired with rhodanine staining of histologic liver sections is the current gold standard to assess hepatic copper and diagnose CAH in dogs (8).

## 9 Diagnosis of Wilson disease (WD)

The diagnostic evaluation of WD in humans also begins with a detailed medical history including family history and a thorough physical examination. Symptoms of ocular disease should be interrogated with a slit-lamp examination or optical tomography to evaluate for KF rings and other ocular abnormalities. As also recommended by European Association for the Study of Liver Disease guidelines (42), a neurologic evaluation should be performed and possibly an MRI because MRI findings are detected in 40% of patients with the hepatic form of the disease (92). A complete blood count, biochemistry panel, and urinalysis are performed to identify evidence of hemolytic anemia, elevated transaminase activity, and renal injury associated with WD. Other non-invasive baseline tests that are performed in the diagnosis of WD include measurement of serum ceruloplasmin, 24-h urinary copper excretion, and relative exchangeable copper (REC) although no single test is reliable for diagnosis. Interpretation of liver histopathology with hepatic copper quantification is considered the most reliable diagnostic method (4).

### 9.1 Serum ceruloplasmin

Human patients with WD are expected to have decreased plasma ceruloplasmin because of the inability of ATP7B to incorporate copper into apoceruloplasmin to produce holoceruloplasmin. However, several possible factors can affect serum ceruloplasmin causing false positive or negative results. Inflammation, hyperestrogenemia, or oral contraceptives are associated with increased ceruloplasmin levels and diseases such as aceruloplasminemia, protein-losing enteropathy, neurologic disorders such as cervical dystonia, other liver diseases, and copper deficiency will lower serum ceruloplasmin. Additionally, the available immunologic assays can overestimate ceruloplasmin concentration compared with the oxidative assays. Generally, a ceruloplasmin concentration < 20 mg/dL is considered consistent with WD although a concentration < 10 mg/dL would be more supportive of a diagnosis (7, 37, 42).

### 9.2 Urinary copper excretion

Elevated 24-h urinary copper excretion is expected in patients with WD. The test must be performed in containers that are free of copper to prevent contamination. A level > 100 μg/24 h in a symptomatic patient would be typical for WD, but any result > 40 μg/24 h (upper limit of normal) could be consistent with WD. Urinary copper increases because of increased circulating non-ceruloplasmin bound copper. However, cholestatic liver disease and renal disease increase urinary copper excretion as well and confound the interpretation of the test result (39–41).

### 9.3 Relative exchangeable copper (REC) and non-ceruloplasmin bound copper (NCC)

A novel biomarker of WD that shows high diagnostic accuracy for WD is relative exchangeable copper (REC). Relative exchangeable copper (REC) is the ratio of exchangeable copper (CuEXC) to total serum copper. Exchangeable copper (CuEXC) is a measurement of bioavailable non-ceruloplasmin bound copper. It is measured by using ethylenediaminetetraacetic acid (EDTA) to bind copper followed by an ultrafiltration step and copper quantification using FAAS. The diagnostic performance of REC in the detection of WD is high with a sensitivity of 100% and specificity of 99.6% when REC is ≥ 13.8% (93). Instead of measuring bioavailable copper using EDTA and ultrafiltration, a copper speciation method can be utilized using strong anion exchange chromatography coupled with ICP-MS to measure non-ceruloplasmin bound copper (NCC). However, NCC is more commonly used as a monitoring tool to evaluate treated patients (94, 95) while REC is used as a diagnostic tool. Relative exchangeable copper (REC) is currently available for clinical use in Europe, but not in the United States. Non-ceruloplasmin bound copper (NCC) has limited clinical availability as of now.

### 9.4 ATP7B testing

Genotyping of *ATP7B* can assist in confirming the diagnosis of WD if a common pathogenic mutation is identified, but penetrance is incomplete and genotype–phenotype correlation is poor (96–99). Genotyping of *ATP7B* is available via various laboratories, but is expensive. Quantification of ATP7B peptides can also identify WD patients in 92% of ambiguous cases, but the test is mainly used for research at this time (100).

The Leipzig scoring combines the results of clinical symptoms and features (K-F rings, neuropsychiatric symptoms, Coombs negative hemolytic anemia), decreased ceruloplasmin, elevated 24-h urinary copper excretion, elevated hepatic copper concentration, and genotyping of *ATP7B* to determine the likelihood of WD (101). The calculation of this score and REC is recommended to diagnose WD (42). If the diagnosis is questionable with non-invasive testing, a liver biopsy should be pursued to clarify the diagnosis although histopathologic features can vary.

### 9.5 Liver histopathology

**Figure 2:**
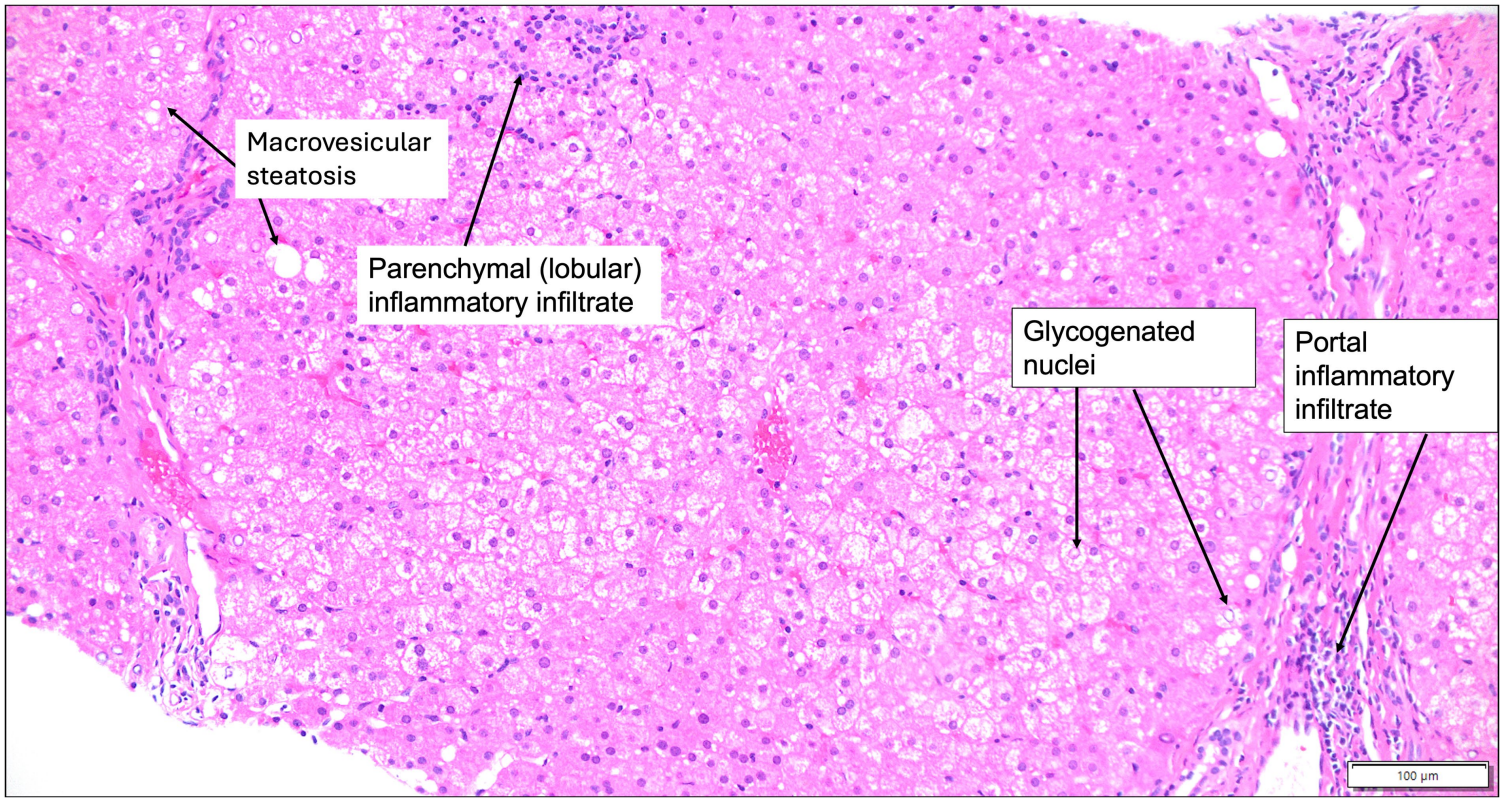
Liver histology of Wilson disease. Liver histology at 200x magnification from a 7-year-old male patient with Wilson disease who presented for cerebellar ataxia, shows mild portal and lobular inflammatory infiltrates that are predominantly lymphocytic, glycogenated hepatocyte nuclei, and minimal macrovesicular steatosis on a hematoxylin and eosin-stained section. Hepatic copper was > 1,000 μg/g dry weight. Images obtained courtesy of Dr. Ameet Thakker.

Histopathologic findings of patients with WD can include steatosis (usually macrovesicular and portal) and acute or chronic hepatitis (lymphocytic to lymphohistiocytic, periportal initially but can progress to panlobular over time) with varying degrees of necrosis depending on the stage and fulminant presentation of the disease (Figure 2). With chronicity, fibrosis can develop portally and then begin to bridge, ultimately resulting in cirrhosis (usually micronodular or mixed) (102). Mallory-Denk bodies (indicative of cell cycle dysregulation) and glycogenated hepatocyte nuclei can also be observed. Hepatic copper quantification should reveal concentrations > 250 μg/g dw whereas histochemical staining with rhodanine lacks sensitivity and specificity for WD (15, 103). Mitochondrial pleomorphism (enlargement, separation of inner and outer membranes, cristae dilation) and dense lysosomal inclusions can be visible on electron microscopy indicative of mitochondrial copper accumulation, dysfunction, and oxidative stress (70, 104). Cirrhosis in patients with WD is associated with the development of hepatocellular carcinoma (105), although this is still debated (106). Dogs with CAH have no known increased risk of hepatocellular carcinoma or other neoplasia. Metabolic dysfunction-associated fatty liver disease or metabolic dysfunction-associated steatohepatitis has not been reported to occur in the dog. A Scottish terrier study on progressive vacuolar hepatopathy, predominantly characterized by excessive glycogen accumulation, has suggested adrenal steroidogenesis and a predisposition to hepatocellular carcinoma in this breed (107). However, while hepatocellular carcinomas in dogs occur with relative frequency, an underlying etiology or underlying liver disease is often not apparent.

## 10 Differences in the tests utilized to diagnose copper-associated chronic hepatitis (CAH) and Wilson disease (WD)

Ceruloplasmin is not measured to clinically assess dogs with CAH because Bedlington terriers with the *COMMD1* mutation have normal to increased ceruloplasmin concentrations (36). This is perhaps because COMMD1 defects do not impact holoceruloplasmin formation unlike *ATP7B* mutations in human WD. Differences in serum copper concentrations and copper-carrying compounds between human and dog might explain why neither serum copper nor ceruloplasmin measurement is helpful in dogs with CAH (34). Absolute total serum copper and exchangeable or non-ceruloplasmin bound copper concentrations in dogs are quantitatively half that of humans. Dogs also rely more on transcuprein/alpha 2 macroglobulin and low molecular weight complexes for copper transport and less on ceruloplasmin or albumin compared to humans (83). Low molecular weight complexes, such as the small copper carrier, could be more useful in the diagnosis of CAH in dogs. Urinary small copper carrier was significantly increased in *ATP7B*^−/−^ compared to *ATP7B*^*+*/−^ and *ATP7B^+/+^* Labrador retrievers although correlations with hepatic copper concentration have not been reported (108). Spot samples of urinary copper excretion can be increased in Bedlington terriers with CAH, but 24-h excretion has not been evaluated (45, 109). Urine copper:zinc ratios correlated with hepatic copper concentrations in Labrador retrievers, but there was overlap between the normal and elevated hepatic copper groups (38). Measurements of NCC or CuEXC have not been performed or published in dogs.

Histopathologic findings between CAH and WD differ in some regards although the diagnosis of both requires demonstrating and quantifying excess hepatic copper. Hepatosteatosis, glycogenated nuclei, and Mallory-Denk bodies are frequently observed in patients with WD (15, 103), but are not routinely noted in dogs with CAH (8, 110). Adipocyte infiltration in hepatocytes has been observed in later stages of disease in Bedlington terriers with CAH, but is not reported in other dogs affected by CAH (13, 19, 111). The lack of functional ATP7B in human patients with WD can dysregulate lipid metabolism in the intestine and liver resulting in hepatosteatosis (112, 113). The consumption of high-fat diets can also result in metabolic dysfunction and hepatosteatosis in human patients with WD (60). Additionally, there is a notable difference in the hepatic copper distribution between the 2 species. Human patients with WD preferentially accumulate copper periportally while dogs with CAH deposit copper centrilobularly. The reason for this difference is unknown but could relate to differences in copper transport mechanisms or disease etiology. Periportal copper deposition might occur because of *ATP7B* mutations in human patients with WD while centrilobular copper deposition might occur because of excess dietary copper in dogs similar to sheep with copper toxicosis (114). Because portal perfusion proceeds from zone 1 (portal) to 2 (midzonal) to 3 (centrilobular), a high copper load might saturate periportal and midzonal handling resulting in downstream overflow and deposition in zone 3 (centrilobular). This proposed mechanism is purely hypothetical though and requires study to determine its relevance and accuracy.

## 11 Treatment, monitoring, and prognosis of copper-associated chronic hepatitis (CAH)

Standard therapy of dogs with CAH involves chelation agents, such as D-penicillamine, and a copper-restricted diet (preferably <0.12 mg/100 kcal of copper). Copper intake from water should also be limited to < 0.1 μg/g. The most common chelating agent used is D-penicillamine, which is typically started at a dose of 10-15 mg/kg orally every 12 h (8). D-penicillamine is a monothiol amino acid that chelates divalent metal ions such as copper by binding with its sulfhydryl group to facilitate urinary copper excretion. It also induces metallothionein to bind and sequester free intracellular copper. D-penicillamine might also have immunomodulatory and antifibrotic properties (115). Administration with food can reduce the bioavailability and therefore, it is optimal to administer D-penicillamine 1 h before or 2 h after a meal (116). Gastrointestinal signs such as vomiting, lack of appetite, and diarrhea are among the most common adverse effects encountered in dogs receiving D-penicillamine. It is often necessary to administer D-penicillamine with a meal or lower the dose to reduce gastrointestinal adverse effects. Gradual dose escalation and administration with anti-emetics can also limit gastrointestinal adverse effects. Less common adverse effects include cutaneous reactions, protein-losing nephropathy, cytopenias (thrombocytopenia, neutropenia) (117), and copper deficiency (115). Studies in Doberman pinschers and Labrador retrievers with CAH show that D-penicillamine successfully reduces hepatic copper and improves the associated histopathologic abnormalities (118, 119). Alternative chelators that have been trialed, but require further study, include trientine (2,2,2, tetramine), 2,3,2-tetramine (120, 121), and ammonium tetrathiomolybdate (122). Adverse effects of trientine and 2,3,2-tetramine appear less compared to D-penicillamine, but chelation also seems to be more rapid and more likely to induce copper deficiency (121). Ammonium tetrathiomolybdate forms a complex with copper and albumin in the blood preventing uptake of copper. Adverse effects of ammonium tetrathiomolybdate in dogs can include gastrointestinal signs and possibly hematologic abnormalities (122). Dietary copper restriction is a life-long therapy for dogs with CAH. In the induction phase of treatment, copper-restricted diets are combined with chelation treatment for at least 6 months to achieve adequate copper reduction in the liver. Dietary copper restriction itself without chelation can reduce hepatic copper, but it is optimal to administer dietary treatment in combination with a chelating agent to more promptly reduce hepatic copper concentrations. After at least 6 months of dietary copper restriction and chelation, the chelating agent can be discontinued or reduced in dose or frequency of administration. Once the chelating agent is discontinued, dietary copper restriction can prevent reaccumulation of copper in some dogs (123, 124). However, other dogs will reaccumulate copper despite copper-restricted diets and therefore need a longer course or potentially life-long chelation in addition to dietary restriction of copper while monitoring for signs of copper deficiency (123). Zinc salts (zinc gluconate or zinc acetate) is a maintenance treatment option that can be used in conjunction with dietary restriction of copper after chelation is complete. Zinc induces intestinal and hepatic metallothionein, which blocks intestinal absorption of copper and facilitates its fecal excretion. Because of the mechanism of action, zinc is slow to reduce hepatic copper and is therefore not used as an induction agent (125). Even if used for maintenance therapy, the benefit of zinc beyond what dietary copper restriction achieves is questionable (126). Zinc is also commonly associated with adverse gastrointestinal effects and monitoring of serum zinc concentrations is required to avoid exceeding concentrations > 800 μg/dL that can result in zinc-induced hemolysis. For the aforementioned reasons, zinc is not commonly used in the treatment of CAH in dogs (8).

Monitoring during treatment of dogs with CAH requires monitoring for adverse effects of medications, but also clinical, biochemical, and histopathologic response. Improvements in serum transaminase activity and liver function parameters can be observed in a subset of cases as soon as 1 month into therapy, but normalization of ALT might not occur even after 6 months of treatment. Laboratory markers are generally insensitive to histopathological improvements (118, 119). Therefore, repeat assessment of liver histopathology and hepatic copper quantification is necessary to prove that hepatic copper reduction and resolution of CAH has occurred and is usually performed 6 months into treatment (118, 119). Diagnostic tools used in human WD such as 24-h urinary copper, serum ceruloplasmin, non-ceruloplasmin bound copper are not utilized in dogs. However, urine copper:zinc ratio could be a viable monitoring tool to assess treatment response because ratios decrease as hepatic copper concentrations decrease in Labrador retrievers treated for CAH (38). Prognosis in affected dogs is generally good to excellent if treatment is initiated in a timely fashion although in cases of acute liver failure or cirrhosis, prognosis is typically worse. Thus, an early and accurate diagnosis and prompt initiation of treatment is crucial to optimizing outcomes because liver transplantation is not feasible in dogs.

## 12 Treatment, monitoring, and prognosis of Wilson disease (WD) in humans

The mainstay of therapy for human patients with WD is lifelong treatment with FDA-approved chelating agents, D-penicillamine or trientine, or with zinc salts. Chelators are typically initiated at low doses and titrated gradually to a target induction dose of 15–20 mg/kg/day, divided into two or three doses, with a maintenance dose of 10–15 mg/kg/day (7, 42). Up to 30% of patients on D-penicillamine must discontinue treatment due to adverse events such as neurologic deterioration, fever, hypersensitivity, cutaneous eruptions or other dermatologic signs, lymphadenopathy, nephrotoxicity with proteinuria and casts, bone marrow toxicity, lupus-like syndromes, myasthenia gravis, and copper or iron deficiency resulting in sideroblastic anemia and hepatic siderosis (127). Trientine is generally better tolerated although it can also result in progression of neurologic signs, arthralgia, muscle cramps, and copper deficiency (128). Trientine is available in two formulations: trientine dihydrochloride which requires refrigeration, and trientine tetrahydrochloride, which is more stable and does not require cold storage. Trientine tetrahydrochloride was non-inferior to D-penicillamine in maintaining copper balance over 1 year in stable WD patients and had fewer treatment-related discontinuations (94). The mechanism of action of chelators is generally believed to be exerted through increased urinary copper excretion. However, radiotracer copper-64 positron emission tomography (PET)/computed tomography(CT) showed that trientine tetrahydrochloride also acts by preventing intestinal copper absorption to a more significant extent than D-penicillamine (129). In addition to a chelating agent, all patients are encouraged to avoid high-copper foods (organ meat, shellfish, nuts, chocolate, mushrooms, non-dairy milk alternatives, soy-based products) and limit copper intake to < 0.9 mg copper/day. Diet alone is insufficient in treating patients with WD (7). In addition, once adequate copper balance is achieved, dietary restrictions are generally considered less relevant than anti-copper medications in maintaining copper homeostasis.

Zinc salts can be used as a sole agent in symptomatic patients, but are generally reserved for those who are asymptomatic, intolerant of chelators, or as maintenance therapy following successful chelation (130, 131) because there are a number of patients that are non-responders to Zn monotherapy (132). Adverse effects of zinc salts mainly include gastrointestinal signs similar to dogs with CAH.

In cases of acute liver failure, liver transplantation is generally recommended (133). Other novel treatments on the horizon for WD include bis-choline tetrathiomolybdate, which is a potent copper-binding agent that is more stable than ammonium tetrathiomolybdate. It forms tripartite complexes with albumin-bound copper to prevent copper uptake (134). Bis-choline tetrathiomolybdate seems particularly useful in treating patients with neurologic WD because it does not cause the drug-related paradoxical worsening of neurologic signs unlike D-penicillamine and trientine (135). Methanobactin, a bacterial peptide from *Methylosinus trichosporium*, binds intrahepatocytic copper and has shown promise in animal models by reversing mitochondrial damage. However, clinical trials in humans are still lacking. *ATP7B* gene therapy, delivered via adeno-associated viral (AAV) vectors, has shown phenotypic rescue in mouse models of WD by restoring hepatocellular copper transport and normalizing hepatic copper and histopathologic abnormalities. Early-phase human trials are ongoing, but challenges remain in vector delivery, durability, and immune reactions to treatment (136).

Dogs with CAH and human patients with WD both exhibit reduced antioxidant capacity (137, 138). As a result, dogs with CAH are often prescribed antioxidant support in the form of S-adenosylmethionine (SAMe) and alpha-tocopherol (vitamin E) despite the limited evidence demonstrating a significant clinical, biochemical, or histological benefit (139). However, mitochondrial lipid peroxidation and decreased hepatic vitamin E concentrations observed in Bedlington terrier dogs with CAH and human patients with WD suggest there might be some therapeutic benefit (6). Furthermore, vitamin E can reduce oxidant injury and lipid peroxidation in rodent models (140). Alpha-lipoic acid is also potentially beneficial in binding copper, restoring redox status, and reversing protein oxidation (141, 142). However, more rigorous clinical trials are required to determine the benefits of antioxidant treatments including vitamin E and alpha-lipoic acid.

Monitoring during therapy entails assessing for adherence to the medication regimen, improvement or progression in clinical signs, such as KF rings, neurologic signs, or signs of portal hypertension, and checking laboratory parameters. Laboratory tests should include a complete blood count to monitor for signs of medication toxicity or copper deficiency, evidenced by cytopenias or sideroblastic anemia with hyperferritinemia, a liver panel to monitor for improvements or normalization in liver enzyme and function tests that should occur by 3 months of treatment, and a urinalysis to monitor for nephrotoxicity caused by D-penicillamine or trientine. Further testing such as coagulation testing or abdominal ultrasound might be required in a patient suspected of or diagnosed with portal hypertension. Copper testing by measuring 24-h urinary copper excretion and NCC can further guide therapy by determining if patients are being overtreated or undertreated. Values for 24-h urinary copper excretion will initially rise with chelation treatment and then decrease during the maintenance phase to 150–500 μg/24 h. Non-ceruloplasmin bound copper (NCC) concentrations can also be measured to monitor adequacy of treatment. Non-ceruloplasmin bound copper (NCC) will initially increase during induction of chelation and then decrease to a range of 5–15 μg/dL during maintenance therapy (143). Measurement of CuEXC and copper speciation are better methods to directly quantify the non-ceruloplasmin bound copper pool without relying on unreliable ceruloplasmin measurements. Thus, both CuEXC (144) and NCC are valuable tools to monitor treated patients with WD (94). However, measurement of CuEXC and copper speciation are not currently available in most clinical practices. Twenty-four hour urinary copper excretion values < 100 μg/24 h indicate overtreatment and can be further supported by observing clinical or laboratory signs consistent with copper deficiency as well as measuring low serum copper, low ceruloplasmin, and NCC concentrations < 5 μg/dL. In contrast, undertreatment will be supported by 24-h urinary copper excretion > 500 μg/24 h and NCC > 15 μg/dL. Causes of undertreatment could be the result of underdosing, poor drug absorption, or nonadherence to the medication or dietary recommendations. Target goals for 24-h urinary copper excretion differ for patients on zinc therapy and 24-h urinary zinc excretion can be used to gauge adherence to treatment (42). Consideration should also be given to assay variability when measuring any of these biomarkers because sample collection, sample processing, assay performance, and analytical methods can all influence results. A repeat liver biopsy could be performed to quantify hepatic copper and assess histopathologic response because the time to response and degree of response can vary amongst patients. A subset of patients will experience progression in fibrosis that might not be detected by laboratory or other non-invasive laboratory tests (145, 146).

Prognosis of WD is significantly affected by delayed diagnosis, the presence of cirrhosis at the time of diagnosis, and poor adherence to treatment (147, 148). A prognostic scoring system (New Wilson Index) can be used to estimate the risk of mortality and need for liver transplantation (149). Early detection is important to improve prognosis.

## 13 Conclusion

Copper-associated chronic hepatitis (CAH) in dogs and Wilson disease (WD) in humans are similar in their hepatic manifestations and underlying pathophysiology. Excess hepatic copper accumulation results in oxidative and mitochondrial stress, hepatocellular death, inflammation, and eventual progression of fibrosis to cirrhosis. Thus, early detection is important to improve prognosis. Wilson disease (WD) is frequently associated with extrahepatic manifestations—most notably neurologic and ocular signs—while CAH in dogs is predominantly a hepatic disease. Extrahepatic involvement in dogs is rare but may include intravascular hemolysis or renal tubular injury. Additionally, notable histopathologic differences exist between the two diseases. The zonal distribution of hepatic copper in CAH is centrilobular while the deposition in WD occurs periportally. Histologic features such as steatosis, Mallory-Denk bodies, and glycogenated nuclei are more frequently observed in WD than in CAH.

Hypotheses that could explain these clinical and histologic differences between CAH and WAD include:

1. Distinct etiologic factors of disease.
2. Shorter lifespan in dogs.
3. Differences in copper transport and handling.

These hypotheses remain untested and require further study, but will be discussed and explained further. CAH and WD are thought to arise from a complex interplay of genetic predispositions, metabolic factors, and environmental exposures, but the specific contributors and their relative influence may vary. For example, high levels of inbreeding in dogs can propagate mutations such as the *COMDD1* deletion, an established cause of CAH in Bedlington terriers, or ATP7B:c.4358G > A, a variably present and expressed mutation in Labrador retrievers and Doberman pinschers with CAH. However, the widespread use of commercial diets with highly bioavailable copper is hypothesized to influence the disease greatly and has likely resulted in the increased prevalence of CAH in both predisposed and non-predisposed breeds. In humans with WD, numerous pathogenic *ATP7B* mutations have been identified, but factors such as maternal environment, dietary choline, and high-fat diets contribute to disease as well. High-fat diets and metabolic dysfunction could contribute to the steatosis observed in patients with WD that is not typically observed in dogs with CAH.

Lifespan might also play a role in disease phenotype. Dogs have shorter lifespans and they may not live long enough to accumulate systemic copper levels that are high enough to produce extrahepatic manifestations. Alternatively, species-specific differences in copper handling may offer a protective effect. For example, healthy dogs can tolerate much higher hepatic Cu concentrations compared to humans and the absolute quantity of non-ceruloplasmin bound copper is much smaller in dogs compared to humans. Furthermore, dogs predominantly transport copper in serum via transcuprein and lower molecular weight complexes such as small copper carrier, whereas humans rely greatly on ceruloplasmin and albumin. These differences might influence systemic copper distribution and tissue toxicity because non-ceruloplasmin bound copper is labile and more prone to uptake by extra-hepatic tissues. The difference in copper-carrying transporters also potentially lessens the utility of measuring ceruloplasmin, CuEXC, NCC, or 24-h urinary copper to diagnose or monitor CAH in dogs, but this requires further study.

Limitations of this review include the selection bias inherent to a narrative rather than a systematic review. Another limitation was the quality of the evidence because the evidence base was largely cross-sectional and retrospective in nature with few prospective studies examining treatment response and long-term outcomes. Another limitation was the overall scope of the review given the smaller number of peer-reviewed articles available studying CAH in dogs compared to WD in humans. Many of the studies of CAH in dogs are limited by small sample sizes or cohorts of particular breeds. Additionally, there has been minimal investigation into biomarkers of CAH. Of the available studies, many have aimed to identify markers associated with elevated hepatic copper rather than specific and sensitive tools to diagnose CAH. Despite these limitations, this manuscript highlighted the shared and divergent features of CAH in dogs and WD in humans and recognized the areas of further research and learning opportunity. Examples of future research directions could include:

- Comparative research into copper metabolism in dogs and humans to prevent extrahepatic manifestations of WD
- Genetic, epigenetic, and dietary assessments to understand the etiologic contributors to CAH
- Exploration and validation of non-invasive biomarkers to improve the diagnosis and monitoring of treatment of CAH and WD
- Investigation of adjunct treatment approaches that mitigate genetic or dietary causes or reduce oxidative stress and mitochondrial dysfunction in the liver

Results and insights can then be translated to ultimately advance the diagnosis and management of copper-associated liver diseases in both veterinary and human medicine.

## Glossary

ALP: Alkaline phosphatase
ALT: Alanine aminotransferase
AST: Aspartate aminotransferase
ATOX1: Copper chaperone antioxidant 1
ATP7A: ATP-ase copper transporting alpha
ATP7B: ATP-ase copper transporting beta
CAH: Copper-associated chronic hepatitis
CCS: Copper chaperone for superoxide dismutase
COMMD1: Copper Metabolism Murr1 Domain-containing 1
CuEXC: Exchangeable copper
DMT1: Divalent metal transporter 1
FAAS: Flame atomic absorption spectroscopy
GGT: Gamma-glutamyl transferase
ICP-MS: Inductively coupled plasma mass spectrometry
MT: Metallothionein
NCC: Non-ceruloplasmin bound copper
REC: Relative exchangeable copper
ROS: Reactive oxygen species
SOD1: Superoxide dismutase
WD: Wilson disease

## References

[1] Amundson Laura A Kirn Brent N Swensson Erik J Millican Allison A Fahey George C. Copper metabolism and its implications for canine nutrition. Transl Anim Sci. (2024) 8:147. doi: 10.1093/tas/txad147 PMC10787350 38221962

[2] Lutsenko S Roy S Tsvetkov P. Mammalian copper homeostasis: physiological roles and molecular mechanisms. Physiol Rev. (2025) 105:441–91. doi: 10.1152/physrev.00011.2024, PMID: 39172219 PMC11918410

[3] Zischka H Lichtmannegger J. Pathological mitochondrial copper overload in livers of Wilson's disease patients and related animal models. Ann N Y Acad Sci. (2014) 1315:6–15. doi: 10.1111/nyas.12347, PMID: 24517326

[4] Penning LC Berenguer M Czlonkowska A Double KL Dusek P Espinós C . A century of progress on Wilson disease and the enduring challenges of genetics, diagnosis, and treatment. Biomedicine. (2023) 11:420. doi: 10.3390/biomedicines11020420, PMID: 36830958 PMC9953205

[5] Kim B-E Turski ML Nose Y Casad M Rockman HA Thiele DJ. Cardiac copper deficiency activates a systemic signaling mechanism that communicates with the copper acquisition and storage organs. Cell Metab. (2010) 11:353–63. doi: 10.1016/j.cmet.2010.04.003, PMID: 20444417 PMC2901851

[6] Sokol RJ Twedt D McKim JM Devereaux MW Karrer FM Kam I . Oxidant injury to hepatic mitochondria in patients with Wilson's disease and Bedlington terriers with copper toxicosis. Gastroenterology. (1994) 107:1788–98. doi: 10.1016/0016-5085(94)90822-2, PMID: 7958693

[7] Schilsky ML Roberts EA Bronstein JM Dhawan A Hamilton JP Rivard AM . A multidisciplinary approach to the diagnosis and management of Wilson disease: 2022 practice guidance on Wilson disease from the American Association for the Study of Liver Diseases. Hepatology. (2022) 82:E41–90. doi: 10.1002/hep.32801, PMID: 36151586

[8] Webster CRL Center SA Cullen JM Penninck DG Richter KP Twedt DC . ACVIM consensus statement on the diagnosis and treatment of chronic hepatitis in dogs. J Vet Intern Med. (2019) 33:1173–200. doi: 10.1111/jvim.15467, PMID: 30844094 PMC6524396

[9] Poldervaart JH Favier RP Penning LC van den Ingh TS Rothuizen J. Primary hepatitis in dogs: a retrospective review (2002-2006). J Vet Intern Med. (2009) 23:72–80. doi: 10.1111/j.1939-1676.2008.0215.x, PMID: 19175724

[10] Johnston AN Center SA McDonough SP Wakshlag JJ Warner KL. Hepatic copper concentrations in Labrador retrievers with and without chronic hepatitis: 72 cases (1980-2010). J Am Vet Med Assoc. (2013) 242:372–80. doi: 10.2460/javma.242.3.372, PMID: 23327181

[11] Strickland JM Buchweitz JP Smedley RC Olstad KJ Schultz RS Oliver NB . Hepatic copper concentrations in 546 dogs (1982-2015). J Vet Intern Med. (2018) 32:1943–50. doi: 10.1111/jvim.15308, PMID: 30294943 PMC6272033

[12] Sandahl TD Laursen TL Munk DE Vilstrup H Weiss KH Ott P. The prevalence of Wilson's disease: an update. Hepatology. (2020) 71:722–32. doi: 10.1002/hep.30911, PMID: 31449670

[13] Dirksen K Fieten H. Canine copper-associated hepatitis. Vet Clin North Am Small Anim Pract. (2017) 47:631–44. doi: 10.1016/j.cvsm.2016.11.011, PMID: 28063745

[14] Ullal TV Lakin S Gallagher B Sbardellati N Abdo Z Twedt DC. Demographic and histopathologic features of dogs with abnormally high concentrations of hepatic copper. J Vet Intern Med. (2022) 36:2016–27. doi: 10.1111/jvim.16580, PMID: 36318874 PMC9708449

[15] Schroeder SM Matsukuma KE Medici V. Wilson disease and the differential diagnosis of its hepatic manifestations: a narrative review of clinical, laboratory, and liver histological features. Ann Transl Med. (2021) 9:1394. doi: 10.21037/atm-21-2264, PMID: 34733946 PMC8506558

[16] Hoffmann G van den Ingh TSGAM Bode P Rothuizen J. Copper-associated chronic hepatitis in Labrador retrievers. J Vet Intern Med. (2006) 20:856–61. doi: 10.1111/j.1939-1676.2006.tb01798.x, PMID: 16955809

[17] Speeti M Eriksson J Saari S Westermarck E. Lesions of subclinical doberman hepatitis. Vet Pathol. (1998) 35:361–9. doi: 10.1177/030098589803500505, PMID: 9754541

[18] Ferenci P Stremmel W Członkowska A Szalay F Viveiros A Stättermayer AF . Age and sex but not ATP7B genotype effectively influence the clinical phenotype of Wilson disease. Hepatology. (2019) 69:1464–76. doi: 10.1002/hep.30280, PMID: 30232804

[19] Twedt DC Sternlieb I Gilbertson SR. Clinical, morphologic, and chemical studies on copper toxicosis of Bedlington terriers. J Am Vet Med Assoc. (1979) 175:269–75. doi: 10.2460/javma.1979.175.03.269, PMID: 500453

[20] Bexfield NH Buxton RJ Vicek TJ Day MJ Bailey SM Haugland SP . Breed, age and gender distribution of dogs with chronic hepatitis in the United Kingdom. Vet J. (2012) 193:124–8. doi: 10.1016/j.tvjl.2011.11.024, PMID: 22225827 PMC3400054

[21] Webb CB Twedt DC Meyer DJ. Copper-associated liver disease in Dalmatians: a review of 10 dogs (1998-2001). J Vet Intern Med. (2002) 16:665–8. doi: 10.1892/0891-6640(2002)016<0665:cldida>2.3.co;2, PMID: 12465762

[22] Thornburg LP Shaw D Dolan M Raisbeck M Crawford S Dennis GL . Hereditary copper toxicosis in West Highland white terriers. Vet Pathol. (1986) 23:148–54. doi: 10.1177/030098588602300207, PMID: 3962081

[23] Ubbink GJ Van den Ingh TS Yuzbasiyan-Gurkan V Teske E Van de Broek J Rothuizen J. Population dynamics of inherited copper toxicosis in Dutch Bedlington terriers (1977-1997). J Vet Intern Med. (2000) 14:172–6. doi: 10.1892/0891-6640(2000)014<0172:pdoict>2.3.co;2, PMID: 10772489

[24] Fieten H Gill Y Martin AJ Concilli M Dirksen K van Steenbeek FG . The Menkes and Wilson disease genes counteract in copper toxicosis in Labrador retrievers: a new canine model for copper-metabolism disorders. Dis Model Mech. (2016) 9:25–38. doi: 10.1242/dmm.020263, PMID: 26747866 PMC4728329

[25] Langlois DK Nagler BSM Smedley RC Yang YT Yuzbasiyan-Gurkan V. ATP7A, ATP7B, and RETN genotypes in Labrador retrievers with and without copper-associated hepatopathy. J Am Vet Med Assoc. (2022) 260:1–8. doi: 10.2460/javma.21.12.0541, PMID: 35482566

[26] Beyzaei Z Mehrzadeh A Hashemi N Geramizadeh B. The mutation spectrum and ethnic distribution of Wilson disease, a review. Mol Genet Metab Rep. (2024) 38:101034. doi: 10.1016/j.ymgmr.2023.101034, PMID: 38149214 PMC10750106

[27] Medici V LaSalle JM. Genetics and epigenetic factors of Wilson disease. Ann Transl Med. (2019) 7:S58. doi: 10.21037/atm.2019.01.67, PMID: 31179295 PMC6531661

[28] Chevalier K Mauget-Faÿsse M Vasseur V Azar G Obadia MA Poujois A. Eye involvement in Wilson's disease: a review of the literature. J Clin Med. (2022) 11:11. doi: 10.3390/jcm11092528, PMID: 35566651 PMC9102176

[29] Watson AD Middleton DJ Ilkiw JE. Copper storage disease with intravascular haemolysis in a Bedlington terrier. Aust Vet J. (1983) 60:305–7. doi: 10.1111/j.1751-0813.1983.tb02815.x, PMID: 6651669

[30] Stremmel W Merle U Weiskirchen R. Clinical features of Wilson disease. Ann Transl Med. (2019) 7:S61. doi: 10.21037/atm.2019.01.20, PMID: 31179298 PMC6531660

[31] Appleman EH Cianciolo R Mosenco AS Bounds ME Al-Ghazlat S. Transient acquired fanconi syndrome associated with copper storage hepatopathy in 3 dogs. J Vet Intern Med. (2008) 22:1038–42. doi: 10.1111/j.1939-1676.2008.0140.x, PMID: 18647161

[32] Gagné JW Wakshlag JJ Center SA Rutzke MA Glahn RP. Evaluation of calcium, phosphorus, and selected trace mineral status in commercially available dry foods formulated for dogs. J Am Vet Med Assoc. (2013) 243:658–66. doi: 10.2460/javma.243.5.658, PMID: 23971845

[33] Center SA Richter KP Twedt DC Wakshlag JJ Watson PJ Webster CRL. Is it time to reconsider current guidelines for copper content in commercial dog foods? J Am Vet Med Assoc. (2021) 258:357–64. doi: 10.2460/javma.258.4.357, PMID: 33539212

[34] Thornburg LP. A perspective on copper and liver disease in the dog. J Vet Diagn Invest. (2000) 12:101–10. doi: 10.1177/104063870001200201, PMID: 10730937

[35] Cedeño Y Miranda M Orjales I Herrero-Latorre C Suárez M Luna D . Serum concentrations of essential trace and toxic elements in healthy and disease-affected dogs. Animals. (2020) 10:1052. doi: 10.3390/ani10061052, PMID: 32570865 PMC7341321

[36] Su LC Ravanshad S Owen CA Jr McCall JT Zollman PE Hardy RM. A comparison of copper-loading disease in Bedlington terriers and Wilson's disease in humans. Am J Phys. (1982) 243:G226–30. doi: 10.1152/ajpgi.1982.243.3.G226, PMID: 7114265

[37] Tapper EB Rahni DO Arnaout R Lai M. The overuse of serum ceruloplasmin measurement. Am J Med. (2013) 126:926.e1–5. doi: 10.1016/j.amjmed.2013.01.039 23953876

[38] Fieten H Hugen S van den Ingh TS Hendriks WH Vernooij JC Bode P . Urinary excretion of copper, zinc and iron with and without D-penicillamine administration in relation to hepatic copper concentration in dogs. Vet J. (2013) 197:468–73. doi: 10.1016/j.tvjl.2013.03.003 23583003

[39] Steindl P Ferenci P Dienes HP Grimm G Pabinger I Madl C . Wilson's disease in patients presenting with liver disease: a diagnostic challenge. Gastroenterology. (1997) 113:212–8. doi: 10.1016/s0016-5085(97)70097-0, PMID: 9207280

[40] Merle U Schaefer M Ferenci P Stremmel W. Clinical presentation, diagnosis and long-term outcome of Wilson's disease: a cohort study. Gut. (2007) 56:115–20. doi: 10.1136/gut.2005.087262, PMID: 16709660 PMC1856673

[41] European Association for the Study of the Liver. European Association for Study of L. EASL clinical practice guidelines: Wilson's disease. J Hepatol. (2012) 56:671–85. doi: 10.1016/j.jhep.2011.11.007 22340672

[42] Socha P Jańczyk W Zanetto A Burra P Czlonkowska A Debray D . EASL-ERN clinical practice guidelines on Wilson’s disease. J Hepatol. (2025) 82:690–728. doi: 10.1016/j.jhep.2024.11.007, PMID: 40089450

[43] Ferenci P Steindl-Munda P Vogel W Jessner W Gschwantler M Stauber R . Diagnostic value of quantitative hepatic copper determination in patients with Wilson's disease. Clin Gastroenterol Hepatol. (2005) 3:811–8. doi: 10.1016/s1542-3565(05)00181-3, PMID: 16234011

[44] Favier RP Spee B Schotanus BA van den Ingh TS Fieten H Brinkhof B . COMMD1-deficient dogs accumulate copper in hepatocytes and provide a good model for chronic hepatitis and fibrosis. PLoS One. (2012) 7:e42158. doi: 10.1371/journal.pone.0042158, PMID: 22879914 PMC3412840

[45] Hyun C Filippich LJ. Inherited canine copper toxicosis in Australian Bedlington terriers. J Vet Sci. (2004) 5:19–28. doi: 10.4142/jvs.2004.5.1.19 15028882

[46] Haywood S Fuentealba IC Foster J Ross G. Pathobiology of copper-induced injury in Bedlington terriers: ultrastructural and microanalytical studies. Anal Cell Pathol. (1996) 10:229–41. PMID: 8798284

[47] Goldfischer S Sternlieb I. Changes in the distribution of hepatic copper in relation to the progression of Wilson's disease (hepatolenticular degeneration). Am J Pathol. (1968) 53:883–901. PMID: 4177374 PMC2013542

[48] Stromeyer FW Ishak G. Histology of the liver in Wilson’s disease: a study of 34 cases. Am J Clin Pathol. (1980) 73:12–24. doi: 10.1093/ajcp/73.1.12, PMID: 7352414

[49] Sternlieb I. Mitochondrial and fatty changes in hepatocytes of patients with Wilson’s disease. Gastroenterology. (1968) 55:354–67., PMID: 5675366

[50] Wu X Mandigers PJJ Watson AL van den Ingh T Leegwater PAJ Fieten H. Association of the canine ATP7A and ATP7B with hepatic copper accumulation in Dobermann dogs. J Vet Intern Med. (2019) 33:1646–52. doi: 10.1111/jvim.15536, PMID: 31254371 PMC6639496

[51] Haywood S Swinburne J Schofield E Constantino-Casas F Watson P. Copper toxicosis in Bedlington terriers is associated with multiple independent genetic variants. Vet Rec. (2023) 193:e2832. doi: 10.1002/vetr.2832, PMID: 37038639

[52] Pindar S Ramirez C. Predicting copper toxicosis: relationship between the ATP7A and ATP7B gene mutations and hepatic copper quantification in dogs. Hum Genet. (2019) 138:541–6. doi: 10.1007/s00439-019-02010-y, PMID: 31062085

[53] Forman OP Boursnell ME Dunmore BJ Stendall N van den Sluis B Fretwell N . Characterization of the COMMD1 (MURR1) mutation causing copper toxicosis in Bedlington terriers. Anim Genet. (2005) 36:497–501. doi: 10.1111/j.1365-2052.2005.01360.x 16293123

[54] Haywood S Boursnell M Loughran MJ Trafford J Isherwood D Liu X . Copper toxicosis in non-COMMD1 Bedlington terriers is associated with metal transport gene ABCA12. J Trace Elem Med Biol. (2016) 35:83–9. doi: 10.1016/j.jtemb.2016.01.015, PMID: 27049130

[55] Wu X den Boer ER Vos-Loohuis M Steenbeek FGV Monroe GR Nijman IJ . Investigation of genetic modifiers of copper toxicosis in Labrador retrievers. Life. (2020) 10:266. doi: 10.3390/life10110266, PMID: 33142854 PMC7693796

[56] Fieten H Hooijer-Nouwens BD Biourge VC Leegwater PAJ Watson AL van den Ingh TSGAM . Association of Dietary Copper and Zinc Levels with hepatic copper and zinc concentration in Labrador retrievers. J Vet Intern Med. (2012) 26:1274–80. doi: 10.1111/j.1939-1676.2012.01001.x, PMID: 22998127

[57] Huster D Kühne A Bhattacharjee A Raines L Jantsch V Noe J . Diverse functional properties of Wilson disease ATP7B variants. Gastroenterology. (2012) 142:947–956.e5. doi: 10.1053/j.gastro.2011.12.048, PMID: 22240481 PMC3461965

[58] Gomes A Dedoussis GV. Geographic distribution of ATP7B mutations in Wilson disease. Ann Hum Biol. (2016) 43:1–8. doi: 10.3109/03014460.2015.1051492, PMID: 26207595

[59] Medici V Weiss KH. Genetic and environmental modifiers of Wilson disease. Handb Clin Neurol. (2017) 142:35–41. doi: 10.1016/b978-0-444-63625-6.00004-5, PMID: 28433108

[60] Sarode GV Mazi TA Neier K Shibata NM Jospin G Harder NHO . The role of intestine in metabolic dysregulation in murine Wilson disease. Hepatol Commun. (2023) 7:247. doi: 10.1097/hc9.0000000000000247 PMC10497250 37695076

[61] Sarode GV Kim K Kieffer DA Shibata NM Litwin T Czlonkowska A . Metabolomics profiles of patients with Wilson disease reveal a distinct metabolic signature. Metabolomics. (2019) 15:43. doi: 10.1007/s11306-019-1505-6, PMID: 30868361 PMC6568258

[62] Zhong HJ Liu AQ Huang DN Zhou ZH Xu SP Wu L . Exploring the impact of gut microbiota on liver health in mice and patients with Wilson disease. Liver Int. (2024) 44:2700–13. doi: 10.1111/liv.16046, PMID: 39037193

[63] Cai X Dai J Xie Y Xu S Liu M. Multi-omics study unravels gut microbiota and metabolites alteration in patients with Wilson's disease. Sci Rep. (2024) 14:21025. doi: 10.1038/s41598-024-71740-5, PMID: 39251728 PMC11384772

[64] Petruzzelli R Catalano F Crispino R Polishchuk EV Elia M Masone A . Prion protein promotes copper toxicity in Wilson disease. Nat Commun. (2025) 16:1468. doi: 10.1038/s41467-025-56740-x, PMID: 39922819 PMC11807206

[65] Mordaunt CE Kieffer DA Shibata NM Członkowska A Litwin T Weiss KH . Epigenomic signatures in liver and blood of Wilson disease patients include hypermethylation of liver-specific enhancers. Epigenetics Chromatin. (2019) 12:10. doi: 10.1186/s13072-019-0255-z, PMID: 30709419 PMC6357467

[66] Sarode GV Neier K Shibata NM Shen Y Goncharov DA Goncharova EA . Wilson disease: intersecting DNA methylation and histone acetylation regulation of gene expression in a mouse model of hepatic copper accumulation. Cell Mol Gastroenterol Hepatol. (2021) 12:1457–77. doi: 10.1016/j.jcmgh.2021.05.020, PMID: 34098115 PMC8487080

[67] Medici V Shibata NM Kharbanda KK LaSalle JM Woods R Liu S . Wilson's disease: changes in methionine metabolism and inflammation affect global DNA methylation in early liver disease. Hepatology. (2013) 57:555–65. doi: 10.1002/hep.26047 22945834 PMC3566330

[68] Medici V Shibata NM Kharbanda KK Islam MS Keen CL Kim K . Maternal choline modifies fetal liver copper, gene expression, DNA methylation, and neonatal growth in the tx-j mouse model of Wilson disease. Epigenetics. (2014) 9:286–96. doi: 10.4161/epi.27110, PMID: 24220304 PMC3962539

[69] Teschke R Eickhoff A. Wilson disease: copper-mediated Cuproptosis, Iron-related Ferroptosis, and clinical highlights, with comprehensive and critical analysis update. Int J Mol Sci. (2024) 25:4753. doi: 10.3390/ijms25094753, PMID: 38731973 PMC11084815

[70] Sternlieb I. Mitochondrial and fatty changes in hepatocytes of patients with Wilson's disease. Gastroenterology. (1968) 55:354–67., PMID: 5675366

[71] Zischka H Einer C. Mitochondrial copper homeostasis and its derailment in Wilson disease. Int J Biochem Cell Biol. (2018) 102:71–5. doi: 10.1016/j.biocel.2018.07.001, PMID: 29997057

[72] Tang D Chen X Kroemer G. Cuproptosis: a copper-triggered modality of mitochondrial cell death. Cell Res. (2022) 32:417–8. doi: 10.1038/s41422-022-00653-7, PMID: 35354936 PMC9061796

[73] Polishchuk EV Merolla A Lichtmannegger J Romano A Indrieri A Ilyechova EY . Activation of autophagy, observed in liver tissues from patients with Wilson disease and from *ATP7B*-deficient animals, protects hepatocytes from copper-induced apoptosis. Gastroenterology. (2019) 156:1173–1189.e5. doi: 10.1053/j.gastro.2018.11.032, PMID: 30452922

[74] Hamilton JP Koganti L Muchenditsi A Pendyala VS Huso D Hankin J . Activation of liver X receptor/retinoid X receptor pathway ameliorates liver disease in Atp7B(−/−) (Wilson disease) mice. Hepatology. (2016) 63:1828–41. doi: 10.1002/hep.28406, PMID: 26679751 PMC4874878

[75] Wooton-Kee CR Jain AK Wagner M Grusak MA Finegold MJ Lutsenko S . Elevated copper impairs hepatic nuclear receptor function in Wilson’s disease. J Clin Invest. (2015) 125:3449–60. doi: 10.1172/JCI78991, PMID: 26241054 PMC4588285

[76] Kalita J Kumar V Misra UK Ranjan A Khan H Konwar R. A study of oxidative stress, cytokines and glutamate in Wilson disease and their asymptomatic siblings. J Neuroimmunol. (2014) 274:141–8. doi: 10.1016/j.jneuroim.2014.06.013, PMID: 25002079

[77] Dev S Dong Y Hamilton JP. Hepatic microtubule destabilization facilitates liver fibrosis in the mouse model of Wilson disease. J Mol Med (Berl). (2025) 103:531–45. doi: 10.1007/s00109-025-02535-y, PMID: 40140071 PMC12078373

[78] Schilsky ML. Wilson disease: diagnosis, treatment, and follow-up. Clin Liver Dis. (2017) 21:755–67. doi: 10.1016/j.cld.2017.06.011, PMID: 28987261

[79] Korman JD Volenberg I Balko J Webster J Schiodt FV Squires RH Jr . Screening for Wilson disease in acute liver failure: a comparison of currently available diagnostic tests. Hepatology. (2008) 48:1167–74. doi: 10.1002/hep.22446, PMID: 18798336 PMC4881751

[80] Svetel M Potrebić A Pekmezović T Tomić A Kresojević N Ješić R . Neuropsychiatric aspects of treated Wilson's disease. Parkinsonism Relat Disord. (2009) 15:772–5. doi: 10.1016/j.parkreldis.2009.01.010 19559640

[81] Stremmel W Weiskirchen R. Therapeutic strategies in Wilson disease: pathophysiology and mode of action. Ann Transl Med. (2021) 9:732. doi: 10.21037/atm-20-3090, PMID: 33987430 PMC8106045

[82] Munk DE Vendelbo MH Kirk FT Rewitz KS Bender DA Vase KH . Distribution of non-ceruloplasmin-bound copper after i.v. (64)cu injection studied with PET/CT in patients with Wilson disease. JHEP Rep. (2023) 5:100916. doi: 10.1016/j.jhepr.2023.100916, PMID: 37886434 PMC10597763

[83] Montaser A Tetreault C Linder M. Comparison of copper binding components in dog serum with those in other species. Proc Soc Exp Biol Med. (1992) 200:321–9. doi: 10.3181/00379727-200-43437, PMID: 1615008

[84] Kemp SD Zimmerman KL Panciera DL Monroe WE Leib MS Lanz OI. A comparison of liver sampling techniques in dogs. J Vet Intern Med. (2015) 29:51–7. doi: 10.1111/jvim.12508, PMID: 25417960 PMC4858056

[85] Johnston AN Center SA McDonough SP Warner KL. Influence of biopsy specimen size, tissue fixation, and assay variation on copper, iron, and zinc concentrations in canine livers. Am J Vet Res. (2009) 70:1502–11. doi: 10.2460/ajvr.70.12.1502, PMID: 19951122

[86] Cole TL Center SA Flood SN Rowland PH Valentine BA Warner KL . Diagnostic comparison of needle and wedge biopsy specimens of the liver in dogs and cats. J Am Vet Med Assoc. (2002) 220:1483–90. doi: 10.2460/javma.2002.220.1483, PMID: 12018374

[87] Lerch K Johnson GF Grushoff PS Sternlieb I. Canine hepatic lysosomal copper protein: identification as metallothionein. Arch Biochem Biophys. (1985) 243:108–14. doi: 10.1016/0003-9861(85)90778-7, PMID: 4062298

[88] Miller AJ Center SA Randolph JF Friesen CH Miller AD Warner KW. Disparities in hepatic copper concentrations determined by atomic absorption spectroscopy, inductively coupled plasma mass spectrometry, and digital image analysis of rhodanine-stained sections in dogs. J Am Vet Med Assoc. (2021) 258:395–406. doi: 10.2460/javma.258.4.395, PMID: 33539202

[89] Dirksen K Roelen YS van Wolferen ME Kruitwagen HS Penning LC Burgener IA . Erythrocyte copper chaperone for superoxide dismutase and superoxide dismutase as biomarkers for hepatic copper concentrations in Labrador retrievers. Vet J. (2016) 218:1–6. doi: 10.1016/j.tvjl.2016.10.007 27938702

[90] Dirksen K Verzijl T van den Ingh TSGAM Vernooij JCM van der Laan LJW Burgener IA . Hepatocyte-derived microRNAs as sensitive serum biomarkers of hepatocellular injury in Labrador retrievers. Vet J. (2016) 211:75–81. doi: 10.1016/j.tvjl.2016.01.010, PMID: 27021912

[91] Roelen YS Spee B van Wolferen ME Fieten H den Boer ER. Serum miR-30b is increased in Labrador retrievers with elevated hepatic copper levels. Vet J. (2025) 311:106335. doi: 10.1016/j.tvjl.2025.106335, PMID: 40054727

[92] Litwin T Gromadzka G Członkowska A Gołębiowski M Poniatowska R. The effect of gender on brain MRI pathology in Wilson's disease. Metab Brain Dis. (2013) 28:69–75. doi: 10.1007/s11011-013-9378-2, PMID: 23315358 PMC3562549

[93] Lorenzen C Dons K García-Solà C Forns X Kirk FT Lynderup EM . Relative exchangeable copper, exchangeable copper and total copper in the diagnosis of Wilson disease. Liver Int. (2025) 45:e70089. doi: 10.1111/liv.70089, PMID: 40198317 PMC11977851

[94] Schilsky ML Czlonkowska A Zuin M Cassiman D Twardowschy C Poujois A . Trientine tetrahydrochloride versus penicillamine for maintenance therapy in Wilson disease (CHELATE): a randomised, open-label, non-inferiority, phase 3 trial. Lancet Gastroenterol Hepatol. (2022) 7:1092–102. doi: 10.1016/s2468-1253(22)00270-9, PMID: 36183738

[95] Solovyev N Ala A Schilsky M Mills C Willis K Harrington CF. Biomedical copper speciation in relation to Wilson's disease using strong anion exchange chromatography coupled to triple quadrupole inductively coupled plasma mass spectrometry. Anal Chim Acta. (2020) 1098:27–36. doi: 10.1016/j.aca.2019.11.033, PMID: 31948584

[96] Alonso-Castellano P Tugores A Mariño Z Olveira A Berenguer M Huarte MP . Low penetrance of frequent ATP7B mutations explains the low prevalence of Wilson disease. Lessons from real-life registries. Dig Liver Dis. (2025) 57:443–9. doi: 10.1016/j.dld.2024.09.002, PMID: 39322449

[97] Gromadzka G Bendykowska M Przybyłkowski A. Wilson's disease-genetic puzzles with diagnostic implications. Diagnostics. (2023) 13:287. doi: 10.3390/diagnostics13071287, PMID: 37046505 PMC10093728

[98] Garbuz M Ovchinnikova E Ovchinnikova A Vinokurova V Aristarkhova Y Kuziakova O . Spectrum of pathogenic variants of the ATP7B gene and genotype-phenotype correlation in eastern Eurasian patient cohorts with Wilson's disease. Biomedicine. (2024) 12:833. doi: 10.3390/biomedicines12122833, PMID: 39767741 PMC11673475

[99] Kluska A Kulecka M Litwin T Dziezyc K Balabas A Piatkowska M . Whole-exome sequencing identifies novel pathogenic variants across the ATP7B gene and some modifiers of Wilson's disease phenotype. Liver Int. (2019) 39:177–86. doi: 10.1111/liv.13967, PMID: 30230192

[100] Collins CJ Yi F Dayuha R Duong P Horslen S Camarata M . Direct measurement of ATP7B peptides is highly effective in the diagnosis of Wilson disease. Gastroenterology. (2021) 160:2367–2382.e1. doi: 10.1053/j.gastro.2021.02.052, PMID: 33640437 PMC8243898

[101] Ferenci P Caca K Loudianos G Mieli-Vergani G Tanner S Sternlieb I . Diagnosis and phenotypic classification of Wilson disease. Liver Int. (2003) 23:139–42. doi: 10.1034/j.1600-0676.2003.00824.x, PMID: 12955875

[102] Fanni D Guido M Gerosa C Vallascas V Moi M Coni P . Liver changes in Wilson's disease: the full spectrum. A report of 127 biopsies from 43 patients. Eur Rev Med Pharmacol Sci. (2021) 25:4336–44. doi: 10.26355/eurrev_202106_26142, PMID: 34227068

[103] Stromeyer FW Ishak KG. Histology of the liver in Wilson’s disease: a study of 34 cases. Am J Clin Pathol. (1980) 73:12–24. doi: 10.1093/ajcp/73.1.12, PMID: 7352414

[104] Gerosa C Fanni D Congiu T Piras M Cau F Moi M . Liver pathology in Wilson's disease: from copper overload to cirrhosis. J Inorg Biochem. (2019) 193:106–11. doi: 10.1016/j.jinorgbio.2019.01.008, PMID: 30703747

[105] Gunjan D Shalimar Nadda N Kedia S Nayak B Paul SB . Hepatocellular carcinoma: an unusual complication of longstanding Wilson disease. J Clin Exp Hepatol. (2017) 7:152–4. doi: 10.1016/j.jceh.2016.09.012 28663680 PMC5478940

[106] van Meer S de Man RA van den Berg AP Houwen RHJ Linn FHH van Oijen MGH . No increased risk of hepatocellular carcinoma in cirrhosis due to Wilson disease during long-term follow-up. J Gastroenterol Hepatol. (2015) 30:535–9. doi: 10.1111/jgh.12716, PMID: 25160780

[107] Cortright CC Center SA Randolph JF McDonough SP Fecteau KA Warner KL . Clinical features of progressive vacuolar hepatopathy in Scottish terriers with and without hepatocellular carcinoma: 114 cases (1980-2013). J Am Vet Med Assoc. (2014) 245:797–808. doi: 10.2460/javma.245.7.797, PMID: 25229531

[108] Gioilli BD Kidane TZ Fieten H Tellez M Dalphin M Nguyen A . Secretion and uptake of copper via a small copper carrier in blood fluid. Metallomics. (2022) 14:mfac006. doi: 10.1093/mtomcs/mfac006 35199838 PMC8962702

[109] Su L Owen C Zollman P. A defect of biliary excretion of copper in copper-laden Bedlington terriers. Am J Phys. (1982) 243:G231–6. doi: 10.1152/ajpgi.1982.243.3.G231

[110] Van den Ingh TSGAM Cullen JM Guy CM Grinwise HF. Morphological classification of parenchymal disorders of the canine and feline liver In: WSAVA standards for clinical and histological diagnosis of canine and feline liver diseases (2021)

[111] Hultgren BD Stevens JB Hardy RM. Inherited, chronic, progressive hepatic degeneration in Bedlington terriers with increased liver copper concentrations: clinical and pathologic observations and comparison with other copper-associated liver diseases. Am J Vet Res. (1986) 47:365–77. doi: 10.2460/ajvr.1986.47.02.365 3954222

[112] Muchenditsi A Yang H Hamilton JP Koganti L Housseau F Aronov L . Targeted inactivation of copper transporter Atp7b in hepatocytes causes liver steatosis and obesity in mice. Am J Physiol Gastrointest Liver Physiol. (2017) 313:G39–49. doi: 10.1152/ajpgi.00312.2016, PMID: 28428350 PMC5538836

[113] Fontes A Pierson H Bierła JB Eberhagen C Kinschel J Akdogan B . Copper impairs the intestinal barrier integrity in Wilson disease. Metabolism. (2024) 158:155973. doi: 10.1016/j.metabol.2024.155973, PMID: 38986805

[114] Borobia M Villanueva-Saz S Ruiz de Arcaute M Fernández A Verde MT González JM . Copper poisoning, a deadly hazard for sheep. Animals. (2022) 12:2388. doi: 10.3390/ani12182388 36139248 PMC9495211

[115] Pugliese M Biondi V Gugliandolo E Licata P Peritore AF Crupi R . D-penicillamine: the state of the art in humans and in dogs from a pharmacological and regulatory perspective. Antibiotics. (2021) 10:648. doi: 10.3390/antibiotics10060648, PMID: 34071639 PMC8229433

[116] Langlois DK Lehner AF Buchweitz JP Ross DE Johnson MB Kruger JM . Pharmacokinetics and relative bioavailability of D-penicillamine in fasted and nonfasted dogs. J Vet Intern Med. (2013) 27:1071–6. doi: 10.1111/jvim.12147, PMID: 23875792

[117] Brooks KV Frederick SW Berryessa NA. D-penicillamine-associated neutropenia in a Doberman pinscher. Can Vet J. (2023) 64:639–42. 37397696 PMC10286144

[118] Mandigers PJ Van Den Ingh TS Bode P Rothuizen J. Improvement in liver pathology after 4 months of D-penicillamine in 5 Doberman pinschers with subclinical hepatitis. J Vet Intern Med. (2005) 19:40–3. doi: 10.1892/0891-6640(2005)19<40:iilpam>2.0.co;2 15715046

[119] Fieten H Dirksen K van den Ingh TS Winter EA Watson AL Leegwater PA . D-penicillamine treatment of copper-associated hepatitis in Labrador retrievers. Vet J. (2013) 196:522–7. doi: 10.1016/j.tvjl.2012.12.013, PMID: 23375251

[120] Twedt DC Hunsaker HA Allen KG. Use of 2,3,2-tetramine as a hepatic copper chelating agent for treatment of copper hepatotoxicosis in Bedlington terriers. J Am Vet Med Assoc. (1988) 192:52–6. 3343179

[121] Allen KG Twedt DC Hunsaker HA. Tetramine cupruretic agents: a comparison in dogs. Am J Vet Res. (1987) 48:28–30. doi: 10.2460/ajvr.1987.48.01.28, PMID: 3826839

[122] Langlois DK Querubin JR Schall WD Nelson NC Smedley RC. Ammonium tetrathiomolybdate treatment of copper-associated hepatopathy in dogs. J Vet Intern Med. (2019) 33:1336–43. doi: 10.1111/jvim.15474, PMID: 30883912 PMC6524386

[123] Fieten H Biourge VC Watson AL Leegwater PA van den Ingh TS Rothuizen J. Nutritional management of inherited copper-associated hepatitis in the Labrador retriever. Vet J. (2014) 199:429–33. doi: 10.1016/j.tvjl.2013.12.017 24439471

[124] Fieten H Biourge VC Watson AL Leegwater PA van den Ingh TS Rothuizen J. Dietary management of Labrador retrievers with subclinical hepatic copper accumulation. J Vet Intern Med. (2015) 29:822–7. doi: 10.1111/jvim.12574, PMID: 25776942 PMC4895432

[125] Brewer GJ Dick RD Schall W Yuzbasiyan-Gurkan V Mullaney TP Pace C . Use of zinc acetate to treat copper toxicosis in dogs. J Am Vet Med Assoc. (1992) 201:564–8. doi: 10.2460/javma.1992.201.04.564, PMID: 1517130

[126] Hoffmann G Jones PG Biourge V van den Ingh TS Mesu SJ Bode P . Dietary management of hepatic copper accumulation in Labrador retrievers. J Vet Intern Med. (2009) 23:957–63. doi: 10.1111/j.1939-1676.2009.0352.x, PMID: 19627473

[127] Medici V Trevisan CP D'Incà R Barollo M Zancan L Fagiuoli S . Diagnosis and management of Wilson's disease: results of a single center experience. J Clin Gastroenterol. (2006) 40:936–41. doi: 10.1097/01.mcg.0000225670.91722.59, PMID: 17063115

[128] Taylor RM Chen Y Dhawan A European Consortium. Triethylene tetramine dihydrochloride (trientine) in children with Wilson disease: experience at King’s College Hospital and review of the literature. Eur J Pediatr. (2009) 168:1061–8. doi: 10.1007/s00431-008-0886-8, PMID: 19066958

[129] Kirk FT Munk DE Swenson ES Quicquaro AM Vendelbo MH Schilsky ML . Effects of trientine and penicillamine on intestinal copper uptake: a mechanistic 64 cu PET/CT study in healthy humans. Hepatology. (2024) 79:1065–74. doi: 10.1097/hep.0000000000000708, PMID: 38088886 PMC11019997

[130] Tang S Bai L Hou W Hu Z Chen X Zhao J . Comparison of the effectiveness and safety of D-penicillamine and zinc salt treatment for symptomatic Wilson disease: a systematic review and meta-analysis. Front Pharmacol. (2022) 13:847436. doi: 10.3389/fphar.2022.847436, PMID: 35370752 PMC8975209

[131] Munk DE Lund Laursen T Teicher Kirk F Vilstrup H Ala A Gormsen LC . Effect of oral zinc regimens on human hepatic copper content: a randomized intervention study. Sci Rep. (2022) 12:14714. doi: 10.1038/s41598-022-18872-8, PMID: 36038585 PMC9424214

[132] Weiss KH Gotthardt DN Klemm D Merle U Ferenci–Foerster D Schaefer M . Zinc monotherapy is not as effective as chelating agents in treatment of Wilson disease. Gastroenterology. (2011) 140:1189–1198.e1. doi: 10.1053/j.gastro.2010.12.034, PMID: 21185835

[133] Dhawan A Taylor RM Cheeseman P De Silva P Katsiyiannakis L Mieli-Vergani G. Wilson's disease in children: 37-year experience and revised king's score for liver transplantation. Liver Transpl. (2005) 11:441–8. doi: 10.1002/lt.20352, PMID: 15776453

[134] Kirk FT Munk DE Swenson ES Quicquaro AM Vendelbo MH Larsen A . Effects of tetrathiomolybdate on copper metabolism in healthy volunteers and in patients with Wilson disease. J Hepatol. (2024) 80:586–95. doi: 10.1016/j.jhep.2023.11.023, PMID: 38081365

[135] Weiss KH Askari FK Czlonkowska A Ferenci P Bronstein JM Bega D . Bis-choline tetrathiomolybdate in patients with Wilson's disease: an open-label, multicentre, phase 2 study. Lancet Gastroenterol Hepatol. (2017) 2:869–76. doi: 10.1016/s2468-1253(17)30293-5, PMID: 28988934

[136] Zeng C Lin Y Lu X Chen S Xia Y Zhang K . Evaluation of efficacy and safety of AAV8-ΔC4ATP7B gene therapy in a mutant mouse model of Wilson's disease. Mol Ther Methods Clin Dev. (2025) 33:101435. doi: 10.1016/j.omtm.2025.101435, PMID: 40104154 PMC11919453

[137] Spee B Arends B van den Ingh TS Penning LC Rothuizen J. Copper metabolism and oxidative stress in chronic inflammatory and cholestatic liver diseases in dogs. J Vet Intern Med. (2006) 20:1085–92. doi: 10.1892/0891-6640(2006)20[1085:cmaosi]2.0.co;2, PMID: 17063700

[138] Gromadzka G Przybyłkowski A Litwin T Karpińska A. Antioxidant capacity is decreased in Wilson's disease and correlates to liver function. Biol Trace Elem Res. (2023) 201:1582–7. doi: 10.1007/s12011-022-03277-5, PMID: 35524917

[139] Twedt D Webb C Tetrick M. The effect of dietary vitamin E on the clinical laboratory and oxidant status of dogs with chronic hepatitis. J Vet Intern Med. (2003) 17:418A

[140] Sokol RJ Mckim JM Devereaux MW. α-Tocopherol ameliorates oxidant injury in isolated copper-overloaded rat hepatocytes. Pediatr Res. (1996) 39:259–63. doi: 10.1203/00006450-199602000-00012, PMID: 8825797

[141] Kabin E Dong Y Roy S Smirnova J Smith JW Ralle M . Α-lipoic acid ameliorates consequences of copper overload by up-regulating selenoproteins and decreasing redox misbalance. Proc Natl Acad Sci USA. (2023) 120:e2305961120. doi: 10.1073/pnas.2305961120, PMID: 37751556 PMC10556618

[142] Smirnova J Kabin E Järving I Bragina O Tõugu V Plitz T . Copper(I)-binding properties of de-coppering drugs for the treatment of Wilson disease. α-lipoic acid as a potential anti-copper agent. Sci Rep. (2018) 8:1463. doi: 10.1038/s41598-018-19873-2, PMID: 29362485 PMC5780470

[143] Pfeiffenberger J Lohse CM Gotthardt D Rupp C Weiler M Teufel U . Long-term evaluation of urinary copper excretion and non-caeruloplasmin associated copper in Wilson disease patients under medical treatment. J Inherit Metab Dis. (2019) 42:371–80. doi: 10.1002/jimd.12046, PMID: 30746719

[144] Ngwanou DH Couchonnal E Parant F Belmalih A Guillaud O Dumortier J . Long-term urinary copper excretion and exchangeable copper in children with Wilson disease under chelation therapy. J Pediatr Gastroenterol Nutr. (2022) 75:e75–80. doi: 10.1097/mpg.0000000000003531, PMID: 35706098

[145] Sini M Sorbello O Sanna F Battolu F Civolani A Fanni D . Histologic evolution and long-term outcome of Wilson's disease: results of a single-center experience. Eur J Gastroenterol Hepatol. (2013) 25:111–7. doi: 10.1097/MEG.0b013e328358f7da, PMID: 23011036

[146] Cope-Yokoyama S Finegold MJ Sturniolo GC Kim K Mescoli C Rugge M . Wilson disease: histopathological correlations with treatment on follow-up liver biopsies. World J Gastroenterol. (2010) 16:1487–94. doi: 10.3748/wjg.v16.i12.1487, PMID: 20333789 PMC2846254

[147] Członkowska A Niewada M Litwin T Kraiński Ł Skowrońska M Piechal A . Seven decades of clinical experience with Wilson's disease: report from the national reference centre in Poland. Eur J Neurol. (2024) 31:e15646. doi: 10.1111/ene.15646, PMID: 36427277 PMC11464408

[148] Beinhardt S Leiss W Stättermayer AF Graziadei I Zoller H Stauber R . Long-term outcomes of patients with Wilson disease in a large Austrian cohort. Clin Gastroenterol Hepatol. (2014) 12:683–9. doi: 10.1016/j.cgh.2013.09.025, PMID: 24076416

[149] Petrasek J Jirsa M Sperl J Kozak L Taimr P Spicak J . Revised King's College score for liver transplantation in adult patients with Wilson's disease. Liver Transpl. (2007) 13:55–61. doi: 10.1002/lt.20920, PMID: 17154398

